# Reported penicillin allergy in Israel: Clinical outcomes and antibiotic costs in a nationwide population-based cohort study

**DOI:** 10.1016/j.jacig.2025.100565

**Published:** 2025-08-28

**Authors:** Shirley Shapiro Ben David, Avner Kantor, Beatriz Hemo, Swetlana Donskoi, Sharon Baruch-Gez, Daniella Rahamim-Cohen, Na’ama Shamir-Stein, Edna Bar-Rason, Alon Y. Hershko

**Affiliations:** a Maccabi Healthcare Services, Tel Aviv, Israel; b Faculty of Medicine, Tel Aviv University, Tel Aviv, Israel; c Department of Medicine, Hadassah Medical Center, Faculty of Medicine, Hebrew University of Jerusalem, Jerusalem, Israel

**Keywords:** Penicillin allergy, health care utilization, adverse drug reaction, drug hypersensitivity

## Abstract

**Background:**

Penicillin allergy (PA) is the most documented drug allergy and is overdiagnosed. Data on medical aspects and expenditure outcomes of PA in the outpatient setting are important for planning delabeling programs.

**Objective:**

We sought to characterize the features of PA on a nationwide level and associated burden on the health care system.

**Methods:**

This is a retrospective, matched cohort study conducted on members of a single health maintenance organization. Medical records of those with documented PA in 2022 were compared with those of matched subjects without allergy based on age group, sex, ethnicity, socioeconomic status, and comorbidities. Outcomes included physician encounters, hospitalizations, death events, antibiotic purchases, and costs.

**Results:**

From a database of 2,602,110 individuals, 96,675 (3.7%) subjects with documented PA were included. Most were females (63.3%), mean age 47.3 ± 22 years, and had medium to high socioeconomic status (85.6%). PA was associated with more encounters with primary care physicians (odds ratio [OR], 1.42; 95% CI, 1.38-1.46; *P* < .001), pediatricians (OR, 1.1; 95% CI, 1.07-1.14; *P* < .001), and secondary care physicians (OR, 1.21; 95% CI, 1.19-1.24; *P* < .001), and increased hospitalizations (OR, 1.12; 95% CI, 1.07-1.17; *P* < .001). Death events were similar in both groups. PA was associated with increased antibiotic purchases per patient (average, 0.93 ± 1.79 vs 0.8 ± 1.58; *P* < .001) at higher costs (8.91 USD vs 6.03 USD, *P* < .01). It exhibited increased use of clindamycin (OR, 5.66; 95% CI, 5.38-5.95; *P* < .001), macrolides (OR, 4.20; 95% CI, 4.08-4.32; *P* < .001), and quinolones (OR, 1.50; 95% CI, 1.44-1.55; *P* < .001).

**Conclusions:**

Reported PA is associated with an increased burden on health care resources but not increased mortality. PA delabeling strategies should improve antibiotic use and costs.

Penicillin is a beta-lactam antibiotic widely used for treating bacterial infections. It is highly effective against many bacterial infections and is also used for prophylaxis.^1^, ^2^, ^3^ Antibiotics classified as penicillins are the most frequently reported allergenic drugs in outpatient settings, with prevalence rates varying from 6% to 25%.^4^^,^^5^ However, most patients with documented penicillin allergy (PA) are unlikely to experience subsequent reactions.^6^^,^^7^ As many as 90% of these patients are diagnosed erroneously, and on evaluation, fewer than 1% of the population manifest allergic responses to penicillin.^6^, ^7^, ^8^, ^9^ Many patients either do not have IgE-mediated reactions or exhibit symptoms of their underlying illness, wrongly attributed to the antibiotic agent.^10^^,^^11^ Furthermore, approximately 80% of patients with a history of a true PA lose their sensitivity after 10 years.^6^

Individuals who are labeled as allergic to penicillin are often prescribed an alternative second-line antibiotic agent, which is frequently less advantageous.^12^^,^^13^ These alternatives may include fluoroquinolones, clindamycin, vancomycin, and cephalosporins, incurring additional costs and possibly decreasing the therapeutic benefit. A further adverse outcome is an increased risk for infections caused by *Clostridioides difficile*, methicillin-resistant *Staphylococcus aureus*, and vancomycin-resistant *enterococcus*.^14^, ^15^, ^16^ Available data, primarily from inpatient settings, indicate that invalidated documentation of PA can result in increased health care expenses, extended hospital stays, and higher mortality rates.^15^, ^16^, ^17^, ^18^

This study aimed to map the prevalence and clinical characteristics of outpatients with records of PA to enable designing of a future health care strategy. We analyzed a database belonging to a large health maintenance organization that provides health care services to approximately one-quarter of the population in the country.

## Methods

### Database

**Fig 1:**
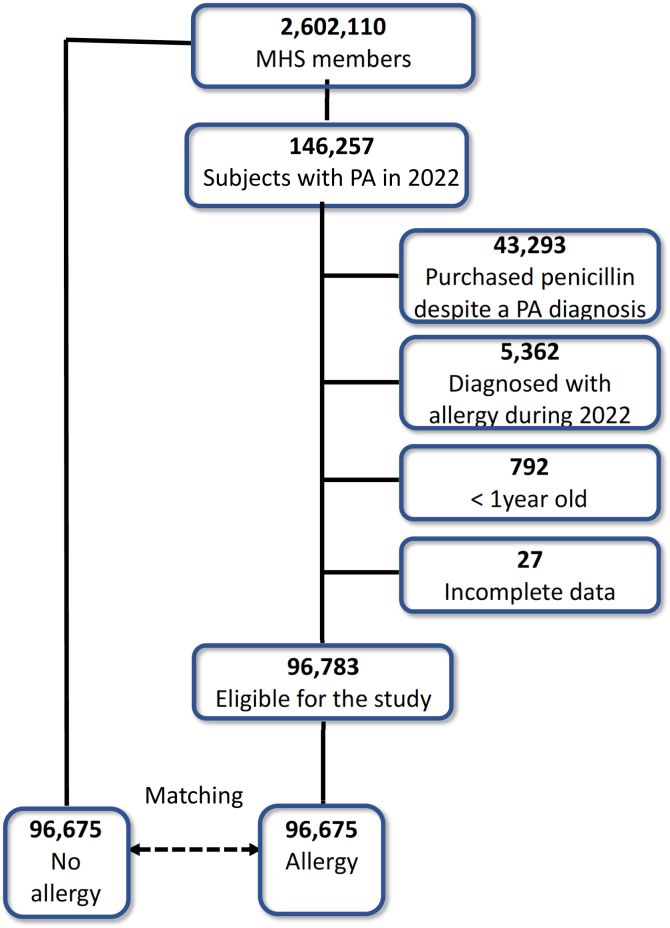
Study design.

This is a retrospective cohort study comparing the records of Maccabi Health Services (MHS) patients with diagnosed PA with those of matched subjects without allergy. MHS is the second-largest health maintenance organization in Israel. It provides health care services to more than 2.6 million members nationwide, equivalent to approximately one-quarter of the Israeli population. It employs more than 6000 physicians who conduct 22 million medical encounters annually. All health care providers within the MHS use a unified electronic health record (EHR) system. All antibiotic purchases require a prescription and are therefore invariably recorded in the EHR system. This study included MHS members from the age of 1 year with a diagnosis of PA (Fig 1). The study period was from January 1 to December 31, 2022. Medical records from the study period were screened for data pertaining to subjects with the PA label and those who are not allergic.

Exclusion criteria: (1) Patients who left the MHS during the study period. (2) Patients with documented penicillin acquisition despite a recorded PA. (3) Patients whose PA diagnosis was first recorded in 2022. The data that were retrieved for the study population included demographic characteristics and socioeconomic status based on patient residence and were categorized according to the definitions of the Central Bureau of Statistics.^19^ In addition, data were collected on medication purchases, drug allergies, chronic illnesses, outpatient visits, diagnoses, and hospitalizations. Chronic illnesses that were retrieved include hypertension, cardiovascular diseases, diabetes, polypharmacy (acquisition of 8 or more drugs), and immunosuppression.^20^ For severe diseases, we used definitions issued by the Israel Ministry of Health.^21^

Assessment of antibiotic costs was based on the number of purchases, using the standard Israel Ministry of Health price list in Israeli currency (New Israeli Shekel-NIS) and are expressed in USD (1 USD = 3.62 NIS).

### Outcomes

We measured the burden of health care utilization among patients with PA in the study period. This was evaluated through the number of outpatient primary care physician encounters, outpatient nonprimary care physician encounters, yearly medical encounters per patient, hospitalizations, hospitalization odds ratio (OR) per patient, all-cause mortality events, number of antibiotic purchases, and costs.

### Statistical analysis

Matching of the allergy and no-allergy groups was done at a ratio of 1:1 according to age group, sex, socioeconomic status group, region of residence, number of chronic illnesses (0, 1-2, 3-4, 5), and the presence of diseases and conditions that may be associated with increased antibiotic use (immunosuppression, inflammatory bowel disease, chronic obstructive pulmonary disease, home care).

Mean frequencies were calculated for each measure of interest. ANOVA was used to compare the mean number of studied measures, controlling for age and chronic illnesses: cardiovascular disease, diabetes, severe disease, cancer, cognitive impairment, osteoporosis, immunosuppression, inflammatory bowel disease, chronic obstructive pulmonary disease, and home care. Logistic regression was conducted to determine the OR of clinic visits or antibiotic purchases, including age and chronic illness of the patients.

Analysis was performed with R, version 4.2.1 (R Foundation for Statistical Computing, Vienna, Austria), and Tidyverse and BroomTidyverse and Broom packages (Posit PBC, Boston, Mass).

### Ethical considerations

The study was conducted with the approval of the Maccabi Internal Review Board (0038-19-MHS). On the basis of retrospective design and the aggregated presentation of findings, the Internal Review Board Committee waived informed consent.

## Results

### Study population

During the study period, 146,257 patients with a documented PA were identified, representing 5.6% of the 2,602,110 MHS member population (Fig 1). Notably, individuals who purchased penicillin after being designated as allergic were excluded from this analysis (n = 43,293). Other subjects who were excluded were those diagnosed during 2022 (n = 5362), infants younger than 1 year (n = 792), and patients with incomplete data (n = 27). Matching for non-PA individuals was obtained for 96,675 patients. Most subjects with PA were women (63%), with a mean age of 47.3 ± 22 years (Table I). The socioeconomic status of 85.6% of subjects with PA was classified as medium to high.

**Table I:** Population characteristics

Characteristic	Allergy	No allergy	*P* value
	N = 96,675	N = 96,675	
Sex: male, n (%)	35,480 (36.7)	35,480 (36.7)	
Age (y), mean ± SD	47.3 ± 22	47.6 ± 22	
Socioeconomic status by deciles, n (%)			
Low, 1-4	13,921 (14.4)	13,921 (14.4)	
Medium, 5-7	43,794 (45.3)	43,794 (45.3)	
High, 8-10	38,960 (40.3)	38,960 (40.3)	
No. of chronic illnesses∗			
None	58,295 (60.3)	58,295 (60.3)	
1-2	22,912 (23.7)	22,912 (23.7)	
3-4	8,991 (9.3)	8,991 (9.3)	
5+	6,574 (6.8)	6,574 (6.8)	
Antibiotic-associated diseases∗	7,734 (8)	7,734 (8)	
Comorbidities, n (%)			
Hypertension	23,782 (24.6)	24,845 (25.7)	<.001
Osteoporosis	12,471 (12.9)	13,341 (13.8)	<.001
Heart disease	10,634 (11)	11,698 (12.1)	<.001
Diabetes	10,441 (10.8)	12,084 (12.5)	<.001
Oncology	9,668 (10)	10,828 (11.2)	<.001
Kidney disease	9,184 (9.5)	11,021 (11.4)	<.05
Polypharmacy	2,224 (2.3)	2,320 (2.4)	<.05

∗ Immunosuppression, inflammatory bowel disease, chronic obstructive pulmonary diseases, and home care.

**Fig 2:**
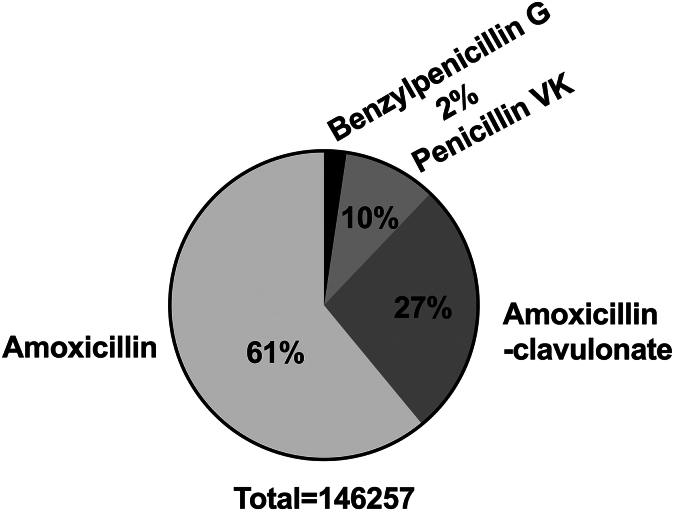
Characteristics of penicillin-related antibiotic use. The total number of penicillin-related regimens that were administered to the PA group before the study period is represented by the relative proportion of each drug.

The most frequently used penicillin-based agent was amoxicillin (61%), followed by amoxicillin-clavulanate (27%), penicillin VK (10%), and benzylpenicillin G (2%) (Fig 2).

### Medical encounters

Compared with subjects without allergy, subjects with PA demonstrated an increased number of annual visits to physicians from various specialties (Table II). The most notable differences were found in allergy/immunology clinics (2.7% vs 1.6%; OR, 1.71; 95% CI, 1.61-1.83), family medicine (88.6% vs 85.1%; OR, 1.42; 95% CI, 1.38-1.46), and pulmonologists (3% vs 2.3%; OR, 1.25; 95% CI, 1.18-1.33). Moreover, those with PAs showed higher annual physician visits per patient rates across most specialties (Table II). Interestingly, the average annual number of PA medical encounters with allergy physicians was slightly lower than that of their matched control.

**Table II:** Annual patient visits∗

Specialty	Total visiting patients, n (%)			Visits per patient, mean ± SD		
	Allergy	No allergy	OR (95% CI)†	Allergy	No allergy	*P* value‡
Family medicine	85,631 (88.6)	82,295 (85.1)	1.42 (1.38-1.46)	9.9 ± 7.7	9.4 ± 7.7	<.001
Dermatology	29,067 (30.1)	26,344 (27.3)	1.15 (1.13-1.17)	1.7 ± 1.3	1.6 ± 1.2	<.001
Gynecology	25,136 (41.1)	23,024 (37.6)	1.10 (1.08-1.13)	3.5 ± 4.0	3.3 ± 3.8	<.001
ENT	16,695 (17.3)	15,035 (15.6)	1.15 (1.12-1.17)	1.6 ± 1.1	1.5 ± 1.1	<.001
Pediatrics	9,170 (79.4)	8,808 (74.5)	1.10 (1.07-1.14)	6.3 ± 5.3	5.9 ± 5.0	<.001
Gastroenterology	9,171 (9.5)	8,272 (8.6)	1.12 (1.08-1.16)	1.6 ± 1.2	1.6 ± 1.2	.362
Urology	6,176 (6.4)	5,698 (5.9)	1.12 (1.08-1.17)	2.1 ± 1.7	2.1 ± 1.7	.884
Pulmonology	2,877 (3)	2,269 (2.3)	1.25 (1.18-1.33)	1.7 ± 1.3	1.7 ± 1.2	.075
Allergy immunology	2,573 (2.7)	1,508 (1.6)	1.71 (1.61-1.83)	1.6 ± 2.1	1.7 ± 2.7	.01

∗ Visits include all face-to-face and virtual and electronic encounters as well as prescription refill requests.

† Analysis is based on logistic regression adjusted for continuous age and chronic illness.

‡ Analysis is based on linear regression adjusted for continuous age and chronic illness.

### Hospitalization and death

**Fig 3:**
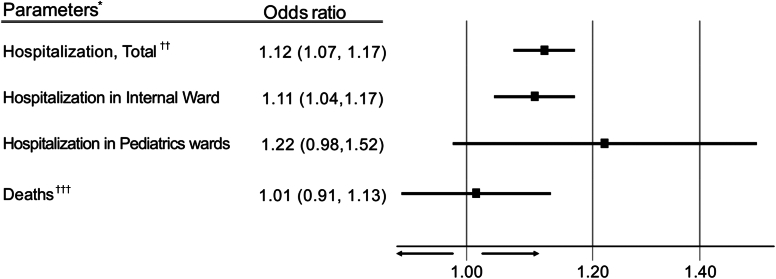
Hospitalization and mortality outcomes in subjects with reported PA. ∗ Analysis is based on logistic regression adjusted for continuous age and chronic illness. †Total admissions include the departments of internal medicine, geriatric medicine, gynecology, urology, ear nose and throat, pediatrics, dermatology, pulmonology, and critical care. ‡All-cause mortality events during 2022.

The absolute number of hospitalizations did not differ between the PA and nonallergy groups (n = 5569 and n = 5573, respectively). However, using logistic regression, PA was shown to increase the risk of hospitalization (OR, 1.12; 95% CI, 1.07-1.17) along with increased admission to the Internal Medicine ward (OR, 1.11; 95% CI, 1.04-1.17) (Fig 3). Conversely, ORs for mortality were similar between the 2 groups (n = 775 and n = 909, respectively; OR, 1.01; 95% CI, 0.91-1.13).

### Antibiotic purchases and costs

**Fig 4:**
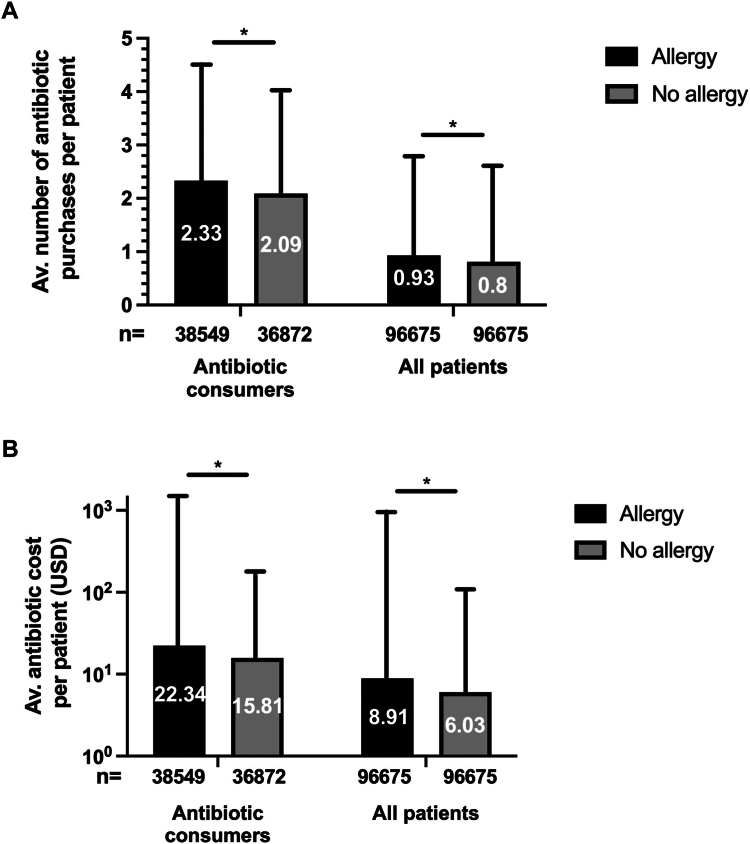
Annual antibiotic purchases and related costs. **A,** Average and SD of antibiotic purchases per patient comparing subjects with allergy and matched subjects without allergy. **B,** Average and SD of antiobiotic costs expressed in US dollars. ∗*P* < .001.

The average rate of annual antibiotic purchases per patient was substantially higher among those with PA than among their matched controls (0.93 ± 1.79 vs 0.80 ± 1.58, *P* < .001) (Fig 4, *A*). Accordingly, among patients who used antibiotics, those with PA purchased more antibiotics than their matched controls without allergy (2.33 ± 2.19 vs 2.09 ± 1.96, *P* < .001).

Furthermore, the average cost of antibiotics was higher among the PA group (8.91 USD vs 6.03 USD, *P* < .001), as well as among patients who purchased antibiotics (22.34 USD compared with 15.81 USD, *P* < .001) (Fig 4, *B*).

**Fig 5:**
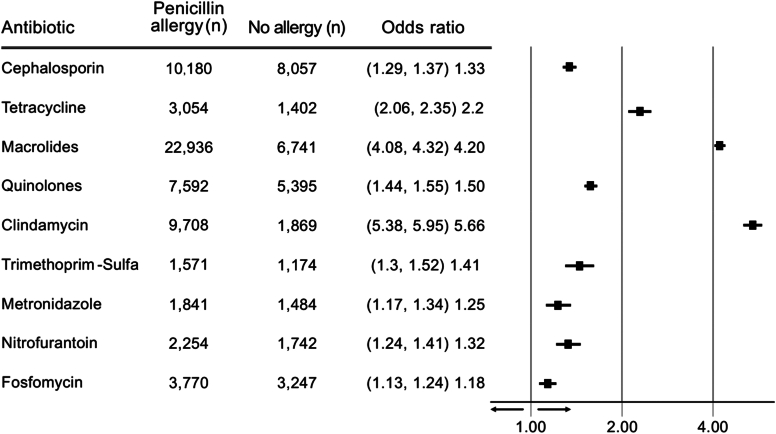
The use of alternative antibiotics in patients with PA. Analysis was based on logistic regression adjusted for continuous age and chronic illnesses.

Compared with the group without allergy, the PA group used more clindamycin (OR, 5.66; 95% CI, 5.38-5.95), macrolides (OR, 4.20; 95% CI, 4.08-4.32), quinolones (OR, 1.50; 95% CI, 1.44-1.55), and other antibiotics (Fig 5).

## Discussion

This study analyzes a large cohort of outpatients with reported PA. The records for this work were retrieved from the MHS database, the health care provider of approximately one-quarter of the Israeli population. Our investigation aimed to expose aspects related to the characteristics and costs of PA in the outpatient setting nationally.

Our data show that 5.63% of the MHS population is designated as allergic to penicillin. These subjects are inclined to use health care services more than matched individuals without allergy. This includes increased physician encounters, hospitalizations, and higher use of antibiotics at elevated costs.

The prevalence of PA in our work is comparable to that in studies from other countries.^4^^,^^5^ The demographic profile showed that most subjects with PA were females (63.3%) and mostly from medium to high socioeconomic backgrounds (85.6%). This is in line with previously published reports suggesting a sex and socioeconomic bias in allergy reporting.^21^, ^22^, ^23^, ^24^ This pattern may be attributed to greater awareness and a higher tendency to report presumably allergic symptoms.^22^^,^^25^

More than 43,000 patients in our database were labeled as penicillin-allergic but subsequently received penicillin after their documented allergy diagnosis. A likely explanation is that physicians intentionally prescribed penicillin but did not remove incorrect allergy labels from medical records, thus leading to persistent misdiagnosis.^26^^,^^27^ The allergy status of this large group is uncertain because our data show antibiotic purchase but do not indicate the actual rate of antibiotic use. Overlooking of PA labels is less probable due to an alert that appears on the screen when an antibiotic is prescribed to an allergic patient. This will block the computer until a justification is typed, representing a local routine that may not exist in other countries. Implementing structured EHR prompts, or standardized allergy review protocols, could help identify and remove incorrect allergy labels, ultimately promoting safer and more effective antibiotic prescribing.^28^^,^^29^ Interestingly, our data indicate that amoxicillin and amoxicillin-clavulanate comprise the majority of penicillins used in the study population (61% and 27%, respectively). This information can guide the choice of antibiotic in a future intervention project for ruling out false allergy diagnoses. Furthermore, it was previously published that some subjects who are allergic to amoxicillin may tolerate benzylpenicillin G.^30^ However, the latter is rarely prescribed by MHS physicians (2%), and, therefore, it does not seem like a practical alternative in this population.

Interestingly, patients with PA exhibited a high rate of physician consultations even though they did not have more comorbidities than matched subjects without allergy. The association between reporting of PA and increased clinic visits may not be causal. Both could be due to an increased personal inclination to interact with the medical system. The most notable differences were found in allergy/immunology clinics (2.7% vs 1.6%; OR, 1.71; 95% CI, 1.61-1.83; Table II). A possible explanation is that matching between the allergy and nonallergy groups did not include atopic diseases such as asthma and rhinitis and therefore such entities could be more common in the PA group. Therefore, the impact of allergy delabeling on the observed number of medical appointments is not necessarily expected to be advantageous.

Patients with PA were more likely to be hospitalized compared with subjects without allergy (OR, 1.12; *P* < .001). Unlike outpatient appointments, which mostly reflect routine visits or mild medical conditions, hospitalization is more likely to indicate acute and more severe illnesses that warrant admission. Increased hospitalizations represent a potential burden on the health care system, and labeling of false PA could hypothetically alleviate this problem. It should be stressed, however, that there was no difference in mortality between the allergy and no-allergy groups. This observation differs from a previous population-based study showing a 14% increased risk of death.^31^ The difference may be attributed to factors such as younger age, lack of chronic diseases or risk factors in most of the population, and other favorable characteristics in the cohort presented herein.

Patients with PA demonstrated higher antibiotic purchases at a higher cost, with an average of 8.91 USD compared with 6.03 USD in subjects without allergy (ie, 48% increase). The increased cost is likely due to the use of more expensive alternative antibiotics, as observed in other studies in the inpatient setting.^32^ Additional factors that may contribute to this finding are increased numbers of antibiotic courses or prolonged duration of treatment. Furthermore, patients with PA were administered a considerably increased number of non–beta-lactam agents. The leading antibiotics were clindamycin, macrolides, and quinolones. Compared with penicillin, these drugs may cause an increased rate of complications such as *C difficile* infection and bacterial resistance.^8^^,^^15^^,^^32^^,^^33^ Although this outcome was not explored in our study, it warrants further investigation to quantify these complications in the outpatient setting. The increased use of alternative antibiotics may also expose the patients to the risk of additional adverse effects that these medications might entail.^34^^,^^35^ It is reasonable to conclude that the diagnosis of PA per se is a pivotal underlying factor to the increased use of non–beta-lactam agents in these subjects. This outcome is expected to be resolved by the ruling out of false PA, which is likely to reduce antibiotic use and costs.

It should be stressed that previous works reported an adverse role of the PA label in specific infectious episodes, as clearly shown in patients with pneumonia.^36^^,^^37^ Our work, however, aimed to delineate the burden of the PA label on a large population that is nationally covered by a health maintenance organization.

The key strength of this study lies in its uniquely large community-based sample with nationwide representation. This work provides an exclusive overview of the outpatient setting, whereas a large proportion of previously published data refer to in-hospital patients. On the basis of these data, we have initiated a large-scale campaign of protocol-driven PA delabeling in collaboration with the MHS. Direct oral challenges will be administered by nonallergist physicians to low-risk subjects in emergency medicine centers across the country. The main limitation of our study is that the allergy is documented under the title of drug reactions in EHR allergy modules. Common side effects, intolerances, and immune-mediated hypersensitivities are often documented similarly and without confirmation by diagnostic tests.^38^^,^^39^ In addition, the quality of allergy reporting can vary on the basis of clinician expertise and interpretation, producing inconsistent or nonspecific data. Another limitation is inherent to the retrospective design, which relies on existing medical records and may not accurately capture all relevant clinical information.

### Conclusion

This study highlights the epidemiological, clinical, and economic implications of recorded PA in the community on a national level. The increased medical expenses and administration of non–beta-lactam agents associated with PA underscore the need for accurate allergy identification and management. Our data will be useful for devising a delabeling strategy that could reduce the harms of erroneous PA diagnosis.

**Clinical implications**

This account of the multiple aspects of PA-associated burden on the health care system may provide justification and a basis for a future large-scale delabeling project.

## Disclosure statement

Disclosure of potential conflict of interest: The authors declare that they have no relevant conflicts of interest.
